# Cost-effectiveness analysis in primary care research: A practical guide for early-career researchers

**DOI:** 10.4102/phcfm.v17i2.5163

**Published:** 2025-09-30

**Authors:** Akim T. Lukwa, Klaus B. von Pressentin, Robert Mash

**Affiliations:** 1 Department of Family, Community and Emergency Care (FaCE), Faculty of Health Sciences, University of Cape Town, Cape Town, South Africa; 2 Health Economics Unit, School of Public Health and Family Medicine, Faculty of Health Sciences, University of Cape Town, Cape Town, South Africa; 3 Division of Family Medicine and Primary Care, Faculty of Medicine and Health Sciences, Stellenbosch University, Stellenbosch, South Africa

**Keywords:** cost-effectiveness analysis, economic evaluation, primary care research, health systems, low- and middle-income countries, resource allocation, CHEERS, early-career researchers, decision modelling, DALYs, QALYs

## Abstract

Cost-effectiveness analysis (CEA) is an important tool for guiding decisions on resource allocation in primary health care (PHC), particularly in low- and middle-income countries that face constrained budgets and competing health priorities. Despite its potential, many early-career primary care researchers struggle with the theoretical and methodological aspects of CEA. This article aims to build capacity in CEA application by providing an accessible guide. It explains fundamental concepts, describes methodological steps, examines quality standards and illustrates real-world applications through detailed case studies from rural settings in Kenya and South Africa. The objective is to equip emerging researchers with the knowledge and skills to embed economic thinking into primary care research and contribute meaningfully to improving the efficiency and equity of health service delivery.

## Introduction

Health systems across the globe are increasingly challenged by the burden of expanding service needs amid limited resources.^1,2^ Nowhere is this tension more acute than in low- and middle-income countries (LMICs), where primary health care (PHC) systems are often required to deliver comprehensive services under financial and infrastructural constraints.^3^ This is crucial considering the pivotal role of PHC and specifically primary care (PC) towards improving health outcomes, health systems efficiency and health equity.^4^ This is where economic evaluation plays a pivotal role. It offers a systematic framework for comparing the costs and health outcomes of alternative interventions, supporting more efficient and equitable resource allocation.^5^ Economic evaluations are broadly categorised into partial and full evaluations, differing in complexity and scope. Partial evaluations such as cost analysis (or cost-minimisation analysis) assess only the costs of interventions that are assumed to have equivalent effects.^6^

Cost-outcomes analysis extends this by documenting both costs and outcomes, but without comparing across alternatives or calculating value-for-money.^7^ In contrast, full economic evaluations assess both costs and outcomes of two or more interventions, enabling direct comparisons.^5^ These include cost-effectiveness analysis (CEA), which measures costs per unit of outcome (e.g. cost per life-year saved); cost-utility analysis (CUA), which incorporates quality-of-life using metrics like quality-adjusted life years (QALYs) (sometimes collectively referred to as health-adjusted life years, HALYs) or disability-adjusted life years (DALYs); and cost-benefit analysis (CBA), which translates both costs and outcomes into monetary terms to determine net benefit.^5^ Importantly, these methods vary in their analytical complexity, with full evaluations requiring more sophisticated modelling techniques, data inputs and methodological assumptions than partial approaches.

Among the various economic evaluation methods, CEA stands out as a particularly valuable tool for guiding investment decisions in health care. Cost-effectiveness analysis provides a structured approach to comparing alternative interventions not only in terms of their clinical effectiveness but also their economic efficiency, answering the question of whether an intervention offers good value for money relative to current practice.^5,8^ Within primary care, where services often address a broad range of preventive, promotive, treatment-related, rehabilitative and palliative needs,^9^ CEA is a critical tool for informing scalable, sustainable investments across this full spectrum of services. Despite its potential, many primary care researchers lack training in health economics, which contributes to its underutilisation in this domain. The present article seeks to bridge this gap by providing a practical roadmap for conducting CEAs in primary care (PC) settings, with a focus on real-world applicability, contextual relevance and methodological rigour.

## The value of cost-effectiveness analysis

Cost-effectiveness analysis is a methodological approach that evaluates the additional cost required to achieve an additional health outcome when comparing two or more interventions.^10^ In PC, CEA plays an essential role in guiding decisions that directly affect population health outcomes. Its utility spans a wide range of services, including human immunodeficiency virus (HIV) prevention,^11^ chronic disease management,^12^ maternal and child health^13^ and emerging areas such as digital health innovations.^14,15^

Using the World Health Organization’s (WHO) Primary Health Care Measurement Framework,^16^ examples of CEA can be categorised across key system domains. At the input level, CEAs have evaluated investments in digital health technologies,^14,15^ health worker training^17^ and supply chain improvements for essential medicines and diagnostics,^18^ helping determine the economic value of strengthening foundational systems. Within service delivery, CEA has informed decisions around task-shifting models,^19^ integrated chronic disease care^12^ and community-based HIV prevention,^20^ illustrating how changes in the organisation and delivery of care can affect both costs and outcomes.

In terms of clinical governance and resilience, emerging CEAs are beginning to assess the value of quality improvement strategies,^21^ patient safety systems^22^ and adaptive delivery platforms, particularly in contexts such as maternal and child health and during health crises.^23^ Despite these advances, CEAs of system-level interventions such as health information systems, delivery models and quality improvement platforms remain limited in scope. As such, there is a pressing need for PC researchers to expand this evidence base by embedding economic evaluation within implementation science, ensuring that investments are not only effective but also financially sustainable and resilient to future shocks.^24^

## Methodological foundations of cost-effectiveness analysis

The conduct of a CEA requires several interrelated methodological steps, beginning with the articulation of a precise research question. This question should identify the target population, the intervention being assessed, an appropriate comparator, the outcomes of interest and the relevant timeframe.^15^ While the Population/Patient/Problem, Intervention, Comparator, Outcome (PICO) framework highlights the need to specify a target population, in primary care research this often encompasses all individuals regardless of age, gender or disease status.^25^ Researchers should therefore apply the PICO structure in a way that maintains the generalist orientation of primary care while still framing clear and answerable questions. Framing the question carefully ensures that the analysis is responsive to real-world decision-making needs. The selection of an analytical perspective is equally critical, as it dictates which costs and outcomes are included in the evaluation.

Common perspectives include those of the healthcare provider^26,27^ and the patient or society.^27,28^ The provider perspective focuses on the costs borne by health systems or facilities such as medication, staffing and infrastructure.^26,27^ The patient perspective captures out-of-pocket expenses, time lost from work and quality of life impacts.^27,28^ The societal perspective is the broadest,^29^ encompassing both provider and patient costs as well as indirect costs such as productivity losses and long-term social consequences. Selecting the appropriate perspective is crucial, as it shapes which costs and benefits are included in the analysis and ultimately influences the interpretation of value for money in healthcare decisions.^30^ In PC, societal perspectives are increasingly relevant because of the high burden of out-of-pocket costs and time lost accessing care.

The measurement of costs must be systematic and transparent. Bottom-up or ingredient-based, costing approaches are often favoured in PC settings because they allow researchers to document and value each resource component of service delivery.^5,31^ Top-down methods, by contrast, distribute aggregate expenditures across services and may be more suitable for subnational-level (district or provincial) analyses,^5^ where cost data are often captured at higher levels of the health system and need to be allocated across service delivery units. Regardless of the approach, costs should be adjusted for inflation, purchasing power and currency differences, and expressed in a common base year for comparability across time, settings and data sources.

For international comparisons, conversions using Purchasing Power Parity (PPP) are preferred. Purchasing Power Parity accounts for differences in the cost of living between countries by equalising the purchasing power of different currencies, allowing for more accurate cross-country comparisons of economic data.^32,33^ Effectiveness measures in CEAs are typically expressed as natural units such as cases treated, lives saved, life-years gained or in standardised metrics like disability-adjusted life years (DALYs) averted or quality-adjusted life years (QALYs). Disability-adjusted life years combine years of life lost (YLL) because of premature mortality and years lived with disability (YLD), where the latter is adjusted using disability weights. These weights reflect the severity of specific health states on a scale from 0 (full health) to 1 (equivalent to death) and are typically derived from population surveys and expert consensus such as those conducted in the Global Burden of Disease (GBD) studies. In contrast, QALYs incorporate both the length and quality of life by weighting time spent in different health states using preference-based utility values; also ranging from 0 (death) to 1 (perfect health) which are usually obtained from methods like time trade-off (TTO), standard gamble (SG) or visual analogue scales (VAS) in general population or patient samples.^34^

Disability-adjusted life years are particularly relevant in LMIC settings because of their alignment with burden of disease metrics and the availability of standardised disability weights.^35^ Once costs and outcomes are estimated, researchers compute the incremental cost-effectiveness ratio (ICER), which expresses the additional cost per additional unit of health benefit gained from the intervention relative to the comparator.^36^ It should be noted that, while calculating an ICER can involve complex decision–analytic modelling such as Markov Models^37^ (particularly for long-term or chronic conditions), it can also be done using simpler, trial-based methods,^38^ when suitable data are available. The level of complexity and therefore the resources required depends on factors such as the time horizon, nature of the intervention, availability of outcome data and whether long-term extrapolation or sensitivity analyses are needed.

The ICER is then compared to a willingness-to-pay (WTP) threshold to determine whether the intervention is considered cost-effective. Traditionally, many studies have used gross domestic product (GDP)-based thresholds, often set at 1–3 times a country’s per capita GDP, following the WHO’s guidance.^10^ For example, if the ICER falls below this benchmark, the intervention is typically deemed cost-effective. However, more recent literature emphasises context-specific thresholds based on health system opportunity costs,^39^ that is, the health benefits forgone when resources are allocated to the evaluated intervention instead of alternative uses. These thresholds reflect how much health the system typically produces with each money unit spent, offering a more realistic benchmark than GDP-based thresholds. For example, if R5000.00 (South African rands [ZAR]) usually buys one DALY averted, this becomes the implicit cost-effectiveness threshold.^39,40^ These empirically derived thresholds tend to be lower than GDP-based ones and are increasingly preferred for aligning CEA with real-world budget constraints, especially in LMICs.^41^

Given inherent uncertainties in input parameters, sensitivity analysis is an indispensable component of CEA.^42,43^ It helps in assessing how changes in assumptions might affect the results. Deterministic sensitivity analysis, for instance, involves varying one parameter at a time such as the cost of an intervention or the effectiveness rate, to examine how much the outcome changes.^44^ Probabilistic sensitivity analysis (PSA), on the other hand, allows multiple parameters to vary simultaneously, based on defined probability distributions and uses repeated simulations to assess the overall robustness of the findings.^45^ To communicate these results, researchers often use visualisation tools like Cost-Effectiveness Acceptability Curves (CEACs), which show the probability that an intervention is cost-effective at different levels of assumptions.

### Case study 1: The Bridging Income Generation with Group Integrated Care study in western Kenya

An example of CEA in PC is the Bridging Income Generation with Group Integrated Care (BIGPIC) study, which evaluated the cost effectiveness of integrated care models for managing non-communicable diseases in western Kenya.^46^ The study focused on two community-based interventions: group medical visits (GMVs) and microfinance (MF), delivered individually or in combination, to address the complex interplay between socioeconomic and medical barriers to care for hypertension and diabetes. Conducted as a cluster-randomised trial, the study enrolled participants into one of four arms: standard care, GMV alone, MF alone and GMV–MF combined. To evaluate cost effectiveness, the researchers built a model that projected cardiovascular events and related DALYs over a 20-year period.

The model simulated how hypertensive and diabetic individuals transition annually between defined health states: starting from no cardiovascular disease, they could experience a heart attack or stroke, move into states of chronic or severe cardiovascular disease and eventually progress to death. Each health state had an associated cost and DALY value. This model was constructed using *TreeAge*, although *Excel* and *R* can also accomplish this. For a novice, constructing a model like this involves defining the key health states, estimating the likelihood of moving between these states each year (transition probabilities) and assigning costs and health outcomes to each state. The costs were derived from detailed programmatic data and micro-costing techniques. Analyses were conducted from the health system perspective, with both costs and outcomes discounted at a 3% annual rate in accordance with international guidelines.^10^

To illustrate, imagine two patients, Akinyi and Otieno, both diagnosed with hypertension. Under usual care, Akinyi visits her clinic quarterly, receives brief consultations and picks up medications. However, adherence is low because of financial and social barriers. Otieno, on the other hand, participates in a GMV programme where he attends bi-monthly sessions led by a clinician and supported by peers. These sessions provide not only medical guidance but also education, psychosocial support and reminders about lifestyle modification. Over time, Otieno’s blood pressure improves significantly, and his risk of cardiovascular events decreases. The BIGPIC study quantified these differences, showing how group-based care could yield better health outcomes at a manageable additional cost.

The GMV Model had an ICER of $1455.00 (United States dollar [USD]) per DALY averted compared to usual care, while the combined GMV–MF intervention had an ICER of USD 3 235 per DALY averted compared to GMV alone. Given the lack of consensus on Kenya’s cost-effectiveness threshold, these interventions were assessed for cost effectiveness at various WTP thresholds; $1040.00 to $3360.00 per DALY averted, thereby rendering the GMV approach cost-effective and the combined model conditionally acceptable.^46^ Importantly, the BIGPIC study not only generated evidence on the value of integrated care but also influenced policy. The findings informed national and subnational strategies for scaling GMVs and attracted donor interest in expanding similar models across rural PC settings.^46^

### Case study 2: Information Communication Technology-enabled Community-Orientated Primary Care in Limpopo, South Africa

In Limpopo province in South Africa, a new way of delivering PHC was tested. This approach, known as Community-Orientated Primary Care (COPC), used trained community health workers (CHWs) supported by digital technology such as mobile apps and tablets to deliver care directly in people’s homes.^47^ The goal was to bring essential health services closer to where people live, especially in areas far from clinics. To determine whether this model was worth the investment, researchers conducted a CEA. They built a decision model to estimate both the costs of the programme and the health benefits it could produce if rolled out across a district of over 600 000 people.

The ICER was calculated by first estimating the total cost of implementing the COPC Model, which included detailed cost inputs such as salaries for CHW teams, digital infrastructure and operational expenses. These costs were then compared to the costs of the existing PC Model (the comparator). To estimate health outcomes, the authors used a simulation model that projected the number of deaths avoided and life-years gained from implementing the COPC intervention. These health gains were based on improvements in preventive and promotive service delivery as well as better continuity of care. The ICER was then calculated by dividing the additional cost of implementing COPC (compared to the comparator) by the additional life-years gained.

The results were striking. The intervention was estimated to save 994 lives per year and gain over 35 000 life-years, at a cost of just R2668.00 (approximately $180.00) per life-year saved, which is well below South Africa’s commonly used cost-effectiveness threshold.^48^ Even better, it led to reduced demand on local clinics and hospitals, translating into health system savings of over R63 million per year. When economic benefits such as avoided healthcare costs and productivity gains were factored in, the programme returned R3.40 for every R1.00 invested.

### Case study 3: Group medical visits for hypertension in rural Kenya – A within-trial cost-effectiveness evaluation

In western Kenya, a different example of CEA comes from a study that evaluated the effect of GMVs on blood pressure control and patient activation within the trial period itself, rather than through long-term modelling as in the BIGPIC study.^49^ Conducted as a cluster-randomised controlled trial across 24 rural health facilities, this study enrolled individuals with uncontrolled hypertension and followed them over a 12-month period. Participants in the intervention arm attended structured GMVs every 2 months, while the control group received standard clinic-based care.

Unlike Case Study 1,^46^ which modelled long-term cardiovascular outcomes and combined medical and socioeconomic interventions (GMVs with or without microfinance), this analysis was limited to short-term outcomes and direct programmatic costs measured during the trial.^49^ The primary clinical outcome was change in systolic blood pressure (SBP), while economic data were derived from detailed programmatic costs and patient-level resource use. Researchers estimated intervention costs including personnel time, training, facility usage and materials. Healthcare utilisation (e.g. outpatient visits, medication use) was tracked using clinical records and self-reports and converted into cost estimates using local unit prices.

The economic evaluation was conducted from the health system perspective, with no discounting required given the 1-year time frame. The analysis calculated the Incremental Cost-Effectiveness Ratio (ICER) as the additional cost per 1-mmHg reduction in SBP for the GMV arm versus usual care.^49^ The results were both intuitive and policy-relevant: the GMV intervention reduced SBP by an average of 6.4 mmHg at an incremental cost of just $30.00 per 1-mmHg reduction.

This case study illustrates a complementary approach to Case Study 1, demonstrating how a simple, within-trial CEA can be implemented using readily available data, without assumptions about long-term disease progression or DALYs. It provides decision-makers with short-term, trial-based evidence that is distinct from modelled, long-term evaluations.^49^

## Reporting standards and knowledge translation

To maximise impact and reproducibility, CEAs should be reported in accordance with the Consolidated Health Economic Evaluation Reporting Standards (CHEERS) 2022 guidelines.^50^ These standards emphasise the need for transparency in model structure, parameter assumptions, costing methods and treatment of uncertainty. Equally important is the translation of findings into policy-relevant formats. While peer-reviewed publications remain important, many policymakers benefit more directly from policy briefs, infographics, and stakeholder presentations. Inclusion of a budget impact analysis alongside a CEA enhances its utility by addressing the fiscal feasibility of implementing a cost-effective intervention at scale.

## Addressing challenges and leveraging opportunities

Conducting CEAs in PC presents several challenges. Data availability is a frequent constraint, particularly in settings lacking routine cost accounting systems or reliable outcome tracking. Moreover, many PC research teams lack in-house economic expertise, which can limit the methodological rigour and credibility of economic evaluations. However, these challenges are not unconquerable. Collaborative models that embed health economists within interdisciplinary research teams can help build local capacity while ensuring high-quality analyses. International training programmes, regional workshops and open-access software have also reduced barriers to entry for early-career researchers.^51^ The increasing demand from funders and ministries of health for evidence of value for money further incentivises the integration of CEA into PC research portfolios. In addition to informing resource allocation, CEA can also support advocacy for greater investment in family medicine and PHC, by providing evidence to engage stakeholders and influence policy.^52,53^

## Conclusion

Cost-effectiveness analysis represents a powerful tool for guiding health policy and resource allocation in PHC. Its ability to quantify the economic and health impacts of competing interventions makes it uniquely suited to support strategic decision-making in resource-constrained settings. For early-career PC researchers, acquiring skills in CEA offers an opportunity to contribute meaningfully to the design, evaluation and scale-up of interventions that improve population health. The BIGPIC and the ICT-enabled COPC case studies illustrate how methodologically rigorous CEAs can influence both policy and practice. As interest in optimising PC grows globally, the ability to conduct and interpret CEAs will be an increasingly valuable skill set for health researchers and practitioners alike.
